# Reductions in abortion-related mortality following policy reform: evidence from Romania, South Africa and Bangladesh

**DOI:** 10.1186/1742-4755-8-39

**Published:** 2011-12-22

**Authors:** Janie Benson, Kathryn Andersen, Ghazaleh Samandari

**Affiliations:** 1 Vice President, Research and Evaluation Unit, Ipas, P.O. Box 5027, Chapel Hill, NC 27514 USA; 2 Senior Associate, Research and Evaluation Unit, Ipas, P.O. Box 5027, Chapel Hill, NC 27514 USA; 3 Independent Consultant, Research and Evaluation Unit, Ipas, P.O. Box 5027, Chapel Hill, NC 27514 USA

## Abstract

Unsafe abortion is a significant contributor to worldwide maternal mortality; however, abortion law and policy liberalization could lead to drops in unsafe abortion and related deaths. This review provides an analysis of changes in abortion mortality in three countries where significant policy reform and related service delivery occurred. Drawing on peer-reviewed literature, population data and grey literature on programs and policies, this paper demonstrates the policy and program changes that led to declines in abortion-related mortality in Romania, South Africa and Bangladesh. In all three countries, abortion policy liberalization was followed by implementation of safe abortion services and other reproductive health interventions. South Africa and Bangladesh trained mid-level providers to offer safe abortion and menstrual regulation services, respectively, Romania improved contraceptive policies and services, and Bangladesh made advances in emergency obstetric care and family planning. The findings point to the importance of multi-faceted and complementary reproductive health reforms in successful implementation of abortion policy reform.

## Background

Unsafe abortion and inadequate post-abortion care are significant contributors to maternal mortality, which is a major cause of death among women of reproductive age worldwide. An estimated 21.6 million unsafe abortions occurred globally in 2008, many of them in developing countries with highly restrictive abortion laws [1]. Approximately 47,000 women die annually from complications of unsafe abortions, a rate of 220 deaths per 100,000 unsafe abortions [1]. These deaths constitute 13% of maternal mortality worldwide, with proportions as high as 49% in some settings [1,2]. In addition to death, approximately 5 million women in the developing world require hospitalization for complications resulting from unsafe abortions, and these complications can lead to long-term health problems [3].

Unsafe abortions, and associated morbidity, mortality and socioeconomic impact, are not inevitable. Unsafe abortion is more likely in settings where there are strong legal prohibitions or where more liberal laws have not translated into access to safe and comprehensive services. The 82 countries with the most restrictive abortion legislation are also those with the highest incidence of unsafe abortions and abortion mortality ratios [4]. In such settings, women often fear legal reprisal or are otherwise unable to access necessary post-abortion care. An estimated 15-25% of women in need of medical treatment for abortion-related complications do not seek care [3]. In many cases women who do try to access the health care system for post-abortion care are met with stigma and given sub-par medical treatment, further compounding the risk of morbidity and mortality [5].

Restrictive abortion policies can also have reverberating social and economic effects on women, their children and larger communities. By 2007, 67 countries had legislation explicitly permitting legal termination on the grounds of economic or social hardship, recognizing the potential impact of unwanted pregnancy and unsafe abortion on women's socio-economic outcomes [6]. Studies also suggest that children born under abortion bans experience substantial socio-economic adversity such as lower rates of education, poor labor market outcomes, higher incidence of mental health problems and higher dependence on welfare [7,8].

The legal context of abortion can significantly affect the incidence of unsafe abortion and its related health consequences; however, an improvement in legislation alone may be insufficient for producing lasting change. In both India and Zambia, abortion was legalized in the early 1970's, but due to a lack of adequate services and continued procedural barriers, safe abortions remain limited. Although health centers in India were mandated to provide safe abortion services in 1971, a shortage of well-trained physicians and appropriate medical equipment has resulted in continued unsafe abortions and an estimated abortion-related mortality ratio of at 37 per 100,000 live births [9-12]. The Zambian abortion law is among the most liberal on the African continent. However, due to required consent from three registered medical practitioners and a lack of available safe abortion services, many women continue to rely on unsafe, clandestine abortions [9], which contribute to the maternal mortality ratio, currently at 591 deaths per 100,000 live births [13-15]. Only recently has the Zambian government made a commitment to address barriers to safe abortion services [16].

**Figure 1:**
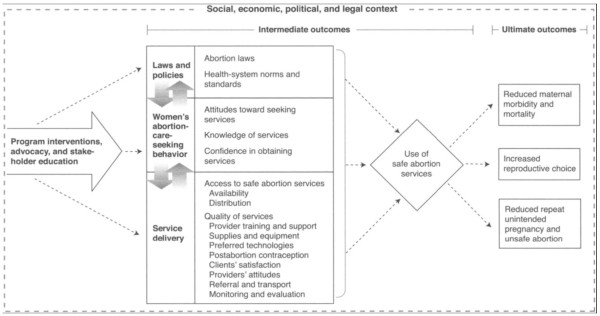
**Conceptual framework for evaluating safe abortion programs (Benson 2005): This figure describes Benson's framework for evaluating safe abortion programs**. Source for this figure is reference [17].

Benson [17] provides a framework positing that abortion law and policy reform has maximal impact on abortion-related maternal mortality when it occurs in conjunction with other supportive programs and services (Figure 1). According to this framework, utilization of safe abortion services, and, ultimately, a reduction in maternal mortality, is best achieved when liberalized abortion policies are supplemented with improved availability and distribution of safe abortion services across a population. Women's knowledge of and attitudes toward abortion, as well as confidence in obtaining services and access to contraception, can also enhance utilization of safe abortion services. The framework suggests that only when these elements coordinate with a loosening of abortion restrictions can real impacts in unsafe abortion and abortion-related mortality be seen. Although Benson's framework provides a theoretical foundation for the paper, this study is not intended to specifically test the components of the model.

Through the use of case studies, this paper explores potential policy and programmatic factors contributing to a decline in abortion-related mortality following abortion policy reform in Romania, South Africa and Bangladesh. These countries were selected on the basis of availability of evidence on abortion-related deaths prior to and following abortion policy change.

## Methods

Through a systematic literature review of peer-reviewed studies, we identified countries where sufficient data and information were available to examine attributes of the reproductive health service delivery context and abortion-related mortality following abortion policy reform. The list of case studies originally included Bangladesh, Cuba, Guyana, South Africa, Romania, Turkey and others. However, only countries for which there was substantial published evidence on trends in abortion related mortality and information on related reproductive health programs were included in the final review; these include Romania, South Africa and Bangladesh.

Articles were identified through a broad literature search including the terms "abortion-related mortality", "maternal mortality" and one or more of the country names. For each article, all possible relevant citations were reviewed for both epidemiologic data on abortion-related mortality and information on policy, service delivery and interventions related to abortion and sexual and reproductive health.

International statistical databases and government/non-governmental (NGO) agency reports for each country were also accessed to aid in the estimation of abortion-related maternal mortality and explanation of relevant programs. Statistical data from pertinent epidemiological sources were extracted and graphed in Excel 2007 to create a representation of trends in abortion-related mortality for each country.

Information on the abortion law reform process and details of supplementary program interventions were gleaned from the peer-reviewed and grey literature. Parameters included: the provision of safe abortion services, availability of safe abortion technologies, training of physicians and mid-level providers in safe abortion care, programs targeting changes in women's and providers' knowledge of and attitudes towards abortion, service guidelines and protocols and access to other services such as family planning or emergency obstetric care.

## Results

The results of this review are presented for three main topics: 1) abortion policy reform; 2) supplemental abortion and reproductive health initiatives; and 3) declines in abortion-related mortality. For Romania and South Africa, abortion-related mortality is compared for the periods prior and subsequent to the policy change. In the case of Bangladesh, where there was no overt abortion law change but rather a change in health system policy towards availability of menstrual regulation (MR), the decline in mortality was measured continuously for the period following the expansion of the MR program in two sub-district areas. Table 1 provides key indicators for reproductive health, socio-economic status and abortion policy for each country.

**Table 1:** Country Profiles in Reproductive Health, Socioeconomic Status and Abortion Policy

	Bangladesh*	Romania	South Africa
**Abortion Rate** **(per 1,000 WRA)**	28.0^a^	27.1^b^**	4.5^c^**
**Modern Contraceptive Prevalence Rate**	47.5%^d^	38.3%^e^	65.0%^f^
**Total Fertility Rate^g^**	2.9	1.3	2.7
**Gross National** **Income per Capita^h^**	$340 (USD)	$10,980 (USD)	$9,560 (USD)
**Female Literacy Rate (ages 15-24)^i^**	41%	98%	94%
**Abortion** **Indications**	Prior to Law Change: To save mother's life Menstrual regulation not permitted After Law Change: Menstrual regulation available on request	Prior to Law Change: Severe mental/physical risk to mother or child Woman over 45 years of age Woman has 5 children under 18 years of age After Law Change: To save the life of the woman To preserve physical health To preserve mental health Rape or incest Fetal impairment Economic or social reasons Available on request	Prior to Law Change: Severe mental/physical risk to mother or child Rape or incest (documentation required) Approval by two physicians After Law Change: To save the life of the woman To preserve physical health To preserve mental health Rape or incest Fetal impairment Economic or social reasons Available on request
**Gestational Age of Abortion**	≤ 10 weeks after last menses	< 12 weeks gestation on request > 12 weeks gestation for therapeutic reasons	< 12 weeks gestation on request > 12 weeks for therapeutic reasons
**Care Provider for Abortion**	Trained paramedic up to 8 weeks gestation Physician up to 10 weeks gestation	Obstetrician-gynecologist only	Medical practitioner, midwife or nurse up to 12 weeks gestation Medical practitioner only after 12 weeks gestation

* Abortion policy refers to the current menstrual regulation policy. **Abortion incidence rates may represent undercounts [78] a [79]; b [24]; c [80]; d [70]; e [35]; f [55]; g [81]; h [82]; i [83].

### Romania

#### Romanian Abortion Policy Reform

As head of the ruling party in 1965, Nikolai Ceauşescu sought to reverse the decline in Romania's population growth by pursuing a rigid and brutally enforced pro-natalist policy. In 1966 and again in 1985, Ceauşescu significantly increased legal restrictions on abortion and banned modern contraceptives [18-22]. As a result, by 1989, Romania had the highest recorded maternal mortality ratio in Europe (170 maternal deaths per 100,000 live births), 87% of which were attributed to abortion complications [19,23,24].

Following the execution of Ceauşescu in 1989, the new Romanian government immediately abolished the restrictive abortion law [19]. This repeal was followed in 1996 by formal legislation which allowed women to freely request a termination of pregnancy within 12 weeks of gestation. Beyond 12 weeks gestation, elective abortion can be performed to preserve the life or physical integrity of the woman. All abortions must be performed by a gynecologist in an approved institution [25].

#### Supplemental Abortion/Reproductive Health Initiatives

In addition to legislative changes, the Romanian government improved family planning and reproductive health (FP/RH) policies and services and increased access to safe abortion. With a grant from the World Bank, the Ministry of Health (MOH) opened 11 family planning referral centers and 230 family planning offices across the country by 1994 [26,27]. It then established the Family Planning and Sexual Education Unit, tasked with procuring contraceptives, developing national FP/RH campaigns, creating FP/RH training curricula for nursing and medical students and training medical practitioners in contraceptive counseling [18,28]. The World Bank loan also provided funding for new abortion medical equipment, such as electric vacuum aspirators. However, without follow-up funding for maintenance, abortion providers reverted to the use of the less-safe dilation and curettage [18].

In 2001, the Romanian government, with support from USAID and John Snow, Inc., established the Romanian Family Health Initiative, (RFHI), which increased access to FP/RH services, particularly for vulnerable populations. RFHI advocated for policy change allowing general practitioners to provide family planning, a task that was previously exclusive to gynecologists. RFHI also assisted the MOH in integrating FP/RH into primary care services [18,28].

In addition, RFHI established the National Family Planning Program (NFPP), to promote the expansion of FP/RH services at the primary health care level through three "pillars." The first pillar consisted of FP/RH training for primary medical care providers, which resulted in 5,105 family practitioners and 3,063 nurses providing contraceptive services by 2006 [28,29]. The second pillar used communication campaigns to increase demand for contraceptive methods among Romanian women, who relied heavily on abortion for fertility control [18,28,30,31]. NFPP's final pillar increased access to contraceptive methods by substantially reducing the cost of methods for vulnerable populations [28,30,32]. In its final years, 2005-2007, the RFHI provided post-abortion care and postpartum contraceptive counseling training to health care providers in 52 public obstetrics-gynecology clinics, which accounted for over half of the abortions performed in Romania [28].

Due to the efforts of the MOH, World Bank and RFPI initiatives, modern contraceptive prevalence steadily increased in Romania from 13.9% in 1993, to 29.5% in 1999 and 38.2% in 2004. Improvements in rural contraceptive prevalence were particularly marked during the years of the RFHI, rising from 20.9% in 1999 to 33.0% in 2004 [33-35]. The abortion rate also declined in recent years to a low of 27.1 per 1,000 women in 2006 [36].

#### Decline in Abortion-Related Mortality

**Figure 2:**
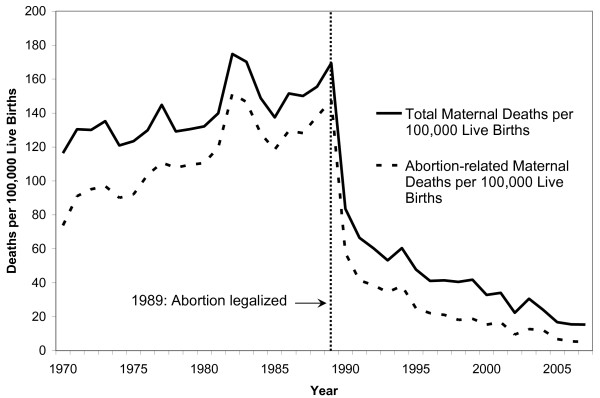
**Total vs. Abortion-related Maternal Deaths per 100,000 Live Births in Romania, 1970-2005: This figure compares the number of abortion-related maternal deaths against total number of maternal deaths per 100,000 live births in Romania prior to and following legalization of abortion in 1989**. Source for this figure is reference [24].

Figure 2 illustrates the dramatic decline in abortion-related mortality following policy reform in 1989. The annual abortion-related mortality ratio dropped from a high of 148 per 100,000 live births in 1989 to 58 per 100,000 in the year immediately following. While 87% of maternal deaths were due to abortion complications in 1989, this proportion dropped to 69% by 1990 [19,24]. Abortion-related mortality continued to decline steadily in subsequent years, as access to safe abortion and comprehensive family planning improved. There was a parallel decline in overall maternal mortality for the same time period, suggesting that the majority of maternal deaths prior to the policy reform were attributable to unsafe abortions. By 2006, the overall maternal mortality ratio dropped to 15 per 100,000 live births, and the abortion-related mortality ratio fell to 5 per 100,000 live births [24].

### South Africa

#### South African Abortion Policy Reform

Despite a purported policy change to overturn an abortion ban in 1975, stringent restrictions perpetuated barriers to accessing safe abortion services (Act No. 72). As a result, the majority of South African women continued to terminate pregnancies through unsafe means, leading to annual unsafe abortions estimates of 120,000-250,000 during the 1975-1996 period [37-39]. By 1994, unsafe abortions had become a long-standing and significant threat to women's health in South Africa [38].

The Choice on Termination of Pregnancy Act (CTOP) of 1996 was enacted by the post-apartheid African National Congress government on October 26, 1996 and implemented on February 1, 1997 [40]. Under the new policy, women have the right to abortion on request during the first 12 weeks of gestation, to protect the health and well-being of the mother or fetus and for reasons of rape/incest between 13-20 weeks (with approval of one physician), and after the 20^th ^week if mother or fetus is at risk of harm (approved by two physicians or one physician and a midwife). These rights were extended to all women in South Africa, including minors; no parental consent is required. Abortion services are provided by trained midwives, nurses or doctors working in licensed facilities, designated by the government as having appropriate equipment and adequate access to emergency care services. All abortion services in the public sector are provided free of charge [41]. An amendment of the CTOP in 2008 increased access to abortion care by further decentralizing control of facility licensing to the provincial level and expanding provider cadres to include trained nurses [42].

#### Supplemental Abortion/Reproductive Health Initiatives

Rapid expansion of abortion services into resource-poor South Africa was achieved through the distribution of low-cost technology (manual vacuum aspiration [MVA] with misoprostol for cervical dilation), supported by external donors. Within the first year of enacting the CTOP, 31,312 legal abortions were performed in designated facilities [43,44]. Despite the rise in safe abortions, the majority of procedures were performed in tertiary hospitals in large urban centers, while abortions in primary health centers, particularly in rural regions, remained limited [45]. By the end of the first year, less than one third of designated hospitals and facilities were providing abortion services, due to lack of trained personnel [46].

To address the training issue, the South African government established the National Abortion Care Programme (NACP) in 1998, which expanded high-quality abortion care services to public clinics and health centers through: 1) training providers in low-cost techniques for safe abortion; 2) training midwives in managing incomplete abortions and performing first trimester abortions with MVA; and 3) training midwives in post-abortion contraceptive counseling. The NACP held national workshops in which physicians or midwives from each province were trained as trainers in abortion care provision and tasked with providing additional training and services in their respective provinces [47]. In addition, the Midwifery Abortion Care Training Programme was developed through local and international private/public health partnerships. These groups established guidelines for the training of nurse midwives, provided national training for registered midwives, and conducted an evaluation of midwives' skill and knowledge in abortion care following training. A national training program for registered midwives commenced in November 1998. By the end of 1999, 103 midwives were certified as abortion providers and prepared to educate other midwives in their home provinces [47-49].

Despite logistical advances, safe abortion advocates were met with some resistance from the medical community on the basis of personal views on abortion. To counteract negative attitudes towards abortion services, over 4,000 nurses, midwives and primary care doctors were enrolled in values clarification workshops designed to facilitate discussion around abortion opinions in the year following the passage of the CTOP [49,50]. Workshop evaluations showed positive changes in attitudes towards new law and an increased interest in providing abortion services [50].

As a result of values clarification workshops, training programs and provider capacity building, by the year 2000, 32% (92/292) of designated facilities were providing services and by 2003, 62% (189/306) of designated facilities offered abortion services [51,52]. By 2006, 17% of *all *community health clinics were authorized to provide abortion services [53].

The decentralization of the health care system following the ANC's ascension in 1994 resulted in parallel improvements in reproductive health services. South Africa has a history of high contraceptive prevalence, but from 1998 to 2003 contraceptive prevalence increased from 61% to 65% among all sexually active women. In particular, the disparities that existed between rural and urban users of contraception all but disappeared from 1998 (67% urban, 54% rural) to 2003 (66% urban, 62% rural) [43,54,55]. In 1997, South Africa also established the National Committee on Confidential Enquiries into Maternal Deaths (NCCEMD), which audits maternal deaths in 200 hospitals across the country and makes recommendations to the Department of Health for improvements in maternal care. Declines in abortion related mortality have been attributed to the work of the NCCEMD and the recommendations stemming from their findings [56]. Based on the confidential reporting system, the NCCEMD is able to ascertain the major causes of maternal deaths in South Africa [43,57].

#### Decline in Abortion-Related Mortality

**Figure 3:**
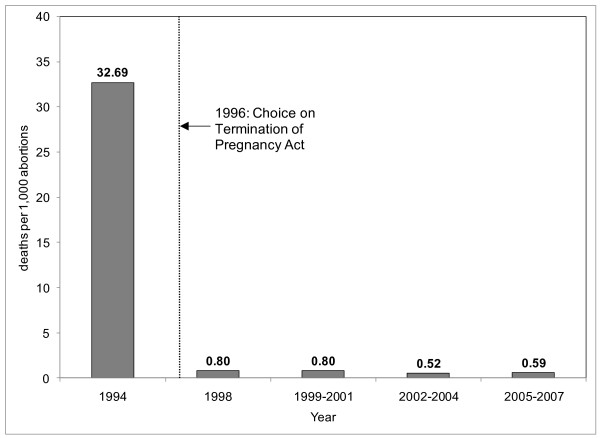
**Abortion-Related Maternal Deaths per 1,000 abortions in South Africa, 1994- 2007: This figure describes the change in abortion-related maternal deaths following the change in South Africa's abortion law in 1996 using a rate of deaths per 1,000 abortions**. The data for this figure were obtained as follows: 1994 data from [38]; 1997 data from [83]; 1999-2001 data from [84,85]; 2002-2004 data from [52] 2005-2007 data from [57] The abortion statistics used for the denominator were retrieved from Health Systems Trust http://www.hst.org.za/healthstats/47/data. It should be noted that some of the abortion statistics for 2005-2007 are incomplete.

The abortion law of 1996 was the first step towards significant reductions in unsafe abortions. The abortion care program and systematic shifts in reproductive health provision was followed by notable declines in abortion-related mortality. Figure 3 shows a decrease in abortion-related deaths in the period following the CTOP. In 1994, complications from unsafe abortion led to 32.69 deaths per 1,000 abortions. By 1998, only 0.80 deaths per 1,000 were reported and this number remained steady or dropped in subsequent years. Furthermore, as compared to 1994, there was a 91% drop in deaths related to unsafe abortion in the 1998-2001 period [58]. By the 2005-2007 period, annual abortion-related deaths accounted for only 3.3% of all maternal deaths [59].

### Bangladesh

#### Abortion Policy Reform

The current abortion law in Bangladesh is based on highly-restrictive mid-19^th ^century British colonial laws, which only permit abortion if the life of the woman is at risk (Penal Code of India of 1860, sections 312-316). Despite the end of colonial rule, local contextual influences resulted in persistence of the abortion ban. However, a menstrual regulation (MR) policy was established by government independence in 1971. Defined as "an interim method to establish a case of non-pregnancy in a woman who is at risk of being pregnant", MR procedures may be performed up to 8 weeks since the last menstrual period (LMP) by a trained paramedic and up to 10 weeks by a trained physician [60,61].

MR services were first introduced in a few urban government family planning clinics in 1974, but by 1979, the government of Bangladesh (GOB) included MR services in the national family planning program. Currently, about 4000 government facilities and 200 NGO clinics offer MR services [61,62]. Private providers offering MR range from trained physicians and paramedic personnel to untrained or traditional practitioners [61].

Widespread access to MR was achieved primarily through training and support of paramedic family welfare visitors (FWV), especially at primary level facilities. Nearly 6,500 FWVs and 8,000 physicians practice MR in government health facilities, with female FWVs exclusively providing MR at the primary health center level [61,63]. Training and support for the MR program is a result of strong NGO-governmental partnerships and continuous efforts from donor organizations to maintain funding for the program. Three local MR NGOs conduct provider training, monitor MR kit distribution and provide MR services in their own sites. In addition to supporting provider salaries, facility space and equipment, the GOB provides services, supervises MR activities in public facilities, and procures and distributes MVA kits [60,62,63].

#### Supplemental Abortion/Reproductive Health Initiatives

In addition to development of the MR program, there were also advances in emergency obstetric care and family planning in Bangladesh. Beginning in 1993, the MOHFW and UNFPA upgraded emergency obstetric care in all 64 maternal and child welfare centers, leading to marked increases in utilization of services [64]. Equipment upgrades and training of medical officers and nurses in treatment of obstetric complications took place from 2000 to 2004 in all 59 district hospitals and 120 of the 400 sub-district hospitals nationally [65]. However, investment in obstetric care remains relatively low in Bangladesh; the maternal mortality ratio at the national level was most recently estimated at 338 per 100,000 in 2008, down from 570 per 100,000 in 2000 [66,67].

Since independence, Bangladesh has also implemented strong FP/RH programs to improve access to and utilization of contraceptive methods. As a result, the total contraceptive prevalence rate for Bangladesh married women of reproductive age increased from 8% in the mid-1970's to 56% in 2007 (48% modern methods) [68-70]. Consequently, the total fertility rate in the same period dropped from 6.3 births per women in 1970-75 to 2.7 births per woman in 2006 [68,70].

In addition to national improvements in reproductive health and maternal care, the International Centre for Diarrhoeal Disease Research, Bangladesh (ICCDR, B) supported rigorous maternal health and obstetric care upgrades and family planning interventions in one-half of the Matlab sub-district of Bangladesh since 1977 [71]. At a comparison research site, the other half of the Matlab sub-district received standard interventions supported by the government and made available nationally [71]. Both the intervention and non-intervention areas have access to regular government-provided MR services.

In the Matlab intervention area, obstetric services include posting of two midwives in each health center, a specialized obstetric clinic and expanded referral and transport for obstetric emergencies [70]. These provisions were made in addition to pre-existing or upgraded national services in maternal health care, as mentioned above. The comparison area in Matlab received routine maternal health services provided by the government, including contraceptive provision and MR, although access to basic obstetric facilities remained limited. Overall, maternal health services in the intervention area are more accessible and of higher quality than those in the comparison area [71]. Evidence from a 2007 study of maternal mortality in Matlab intervention versus comparison areas suggests that overall maternal deaths due to direct obstetric causes in both sites declined in the period from 1976 to 2005, but were substantially lower in the Matlab intervention throughout the study period [71].

The Matlab intervention area also received enhanced FP/RH services. Community health workers visit married women of reproductive age in their homes at regular intervals - initially every two weeks and since 1997, every month -for contraceptive counseling and method delivery and to track demographic and health events. Special MCH centers are also available for FP/RH services [72]. A comparison of contraceptive prevalence and abortion rates from 1979 and 1998 between the intervention and non-intervention areas in Matlab found that abortion rates were significantly lower in the region with better family planning services [72].

#### Decline in Abortion-Related Mortality

Abortion mortality rates were compared between the Matlab intervention area and the comparison site receiving only government programs. Special efforts to track pregnancy-related deaths have occurred since 1976, including interviews with family members [71,72]. This longitudinal surveillance provides a unique opportunity to track and compare long-term trends in maternal mortality in the two areas.

**Figure 4:**
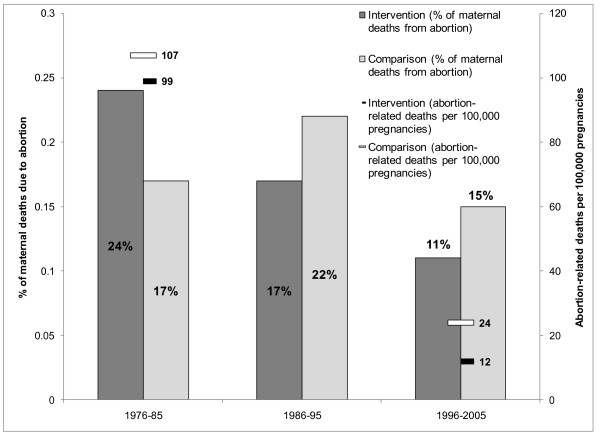
**Proportion of Maternal Deaths due to Abortion and Abortion-Related Deaths in Matlab, Bangladesh, Intervention vs. Comparison areas, 1976 2005: This figure compares the proportion of maternal deaths due to abortion and the number of abortion-related deaths per 100,000 pregnancies in an intervention (Matlab) and comparison area within Bangladesh**. These figures are compared over several blocks of years that include changes in abortion policy. Source for this data is reference [68].

Figure 4 compares the percent of maternal deaths due to abortion in both the intervention and comparison areas in Matlab. In the intervention area, the proportion of maternal deaths due to abortion dropped substantially from 24% in 1976-1985 to 11% in 1996-2005. On the other hand, the proportion of abortion deaths remained fairly stable during the same period in the comparison area: 17% in 1976-1985 and 15% in 1996-2005, with a slight increase to 23% between 1986 and 1995. During the same period, the rate of abortion-related deaths declined in both service areas; however, by 2005, the rate of death in the intervention area (12 per 100,000 pregnancies) was half that of the government area (24 per 100,000 pregnancies) [69]. These comparisons illustrate the importance of the additional FP/RH interventions, above and beyond the menstrual regulation program, in decreasing abortion-related mortality in Bangladesh.

## Discussion

Liberalization of restrictive abortion laws is a critical step in transforming harmful clandestine procedures into safe terminations administered and monitored by trained professionals. However, reforms in abortion policy alone, which are often subject to local contextual influences, may not be sufficient for substantial change in practices or, ultimately, women's health. The countries in this study offer unique insights into the multi-faceted nature of abortion reform.

In all three cases examined here, liberal policies---with broad indications for legal abortion---were followed by strategies to implement abortion services, scale up accessibility and establish complementary reproductive and maternal health services. In Romania, the government and its partners coordinated efforts to train doctors in preferred abortion techniques, expand access to contraception, and address women's dependence on abortion as a method of fertility control. In South Africa, appropriate abortion technologies were rapidly disseminated throughout the country and abortion services were decentralized through training of physicians and midwives. Bangladesh, while not formally changing its abortion law, followed the establishment of a new menstrual regulation policy with national-scale MR training and improvements in family planning and obstetric care. Both South Africa and Bangladesh also made great strides in decentralizing and disseminating abortion care through the training of mid-level providers such as midwives, nurses and paramedical personnel. The examples in this study suggest that a confluence of political will, funding, partnerships between government and NGOs, additional policies supporting change in reproductive services and an overall commitment to improving women's health may contribute to declines in abortion-related mortality. In particular, investments in improved family planning services are essential to preventing unwanted pregnancies, thereby reducing overall demand for abortion. All three cases also demonstrate the important role of research infrastructure in tracking and documenting changes in abortion-related services and outcomes.

Despite their achievements, the long-term experience of these countries highlights the importance of continued support in maintaining gains made in women's health following changes in abortion policies and practices. Romania made early efforts to provide adequate abortion technologies, such as equipment for electric vacuum aspirators; however, with waning financial support, supply lines withered and physicians were forced to revert back to older, less safe methods of inducing abortion such as dilation and curettage. The resultant reduction in the quality of abortion services is compounded with continued gaps in post-abortion care. In South Africa, despite both political will and technical backing to decentralize and disseminate abortion services, safe abortion services are still highly concentrated in urban areas, leaving underserved sites challenged by limitations in providers and supplies. South Africa also continues to struggle with high levels of abortion-related stigma within the medical community. Furthermore, medical abortion drugs and services are only available in the private sector, greatly limiting access to their use. The slight rise in abortion deaths during the period from 2005 to 2007 could be due in part to a decline in abortion services in recent years and to a possible confounding of HIV/AIDS deaths among women of reproductive age in South Africa. In Bangladesh, family planning and obstetric services may not be comprehensive at all sites, leaving an unmet need in some vulnerable populations within the country. The current supply chains for MVA instruments and other supplies needed for MR services are also erratic, leading to periodic interruptions in supplies and re-use of MVA instruments beyond recommended applications. Improved access to high-quality second-trimester abortion services is also needed. General economic, social and cultural barriers may also play an important part in limiting access to comprehensive abortion care in all three study countries.

This review has a few limitations. Due to the reliance on published, available data, we may have omitted other countries with liberalized abortion laws and changes in abortion mortality where data were not available. For this reason, countries such as Turkey and Cuba, where there is anecdotal evidence of a decline in abortion-related mortality after abortion law reform and provision of legal abortion, were not included. Moreover, it was also not possible to include comparison countries where legal reform was not immediately accompanied by supplemental reproductive services. In these cases, such as in Zambia and Ghana, a failure to provide adequate safe abortion and reproductive health services following law reform has resulted in both a dearth of information on attempts to scale-up service provision as well as persistently high maternal and abortion-related death rates [73,74]. In Zambia, despite law change in 1972, there has been little done to promote behavioral or service provision changes until recently [73]. The abortion law in Ghana was liberalized in 1985, but coordinated efforts to improve abortion services and increase awareness of the law have only been implemented as recently as 2006 [75]. In Cambodia, where abortion law reform in 1997 was originally not followed by targeted service provision, efforts are currently underway to increase access to safe abortion and reproductive health care, providing a forthcoming case study on delayed introduction of supplemental abortion and reproductive health services [76]. Other countries with more recent abortion law reform and roll-out of safe abortion include Nepal (2002) and Ethiopia (2005), which will afford opportunities for further research on the impact of policy change on maternal health [77]. Finally, the current review relies primarily on case study analysis methods, which cannot provide casual links between factors in abortion reform and resulting declines in abortion- and does not account for other possible ecological factors that may have contributed to changes in abortion-related mortality in the study countries.

Abortion related mortality continues to play a large role in all-too-high rates of maternal death in many other countries. The solutions for reducing the significant human and economic toll of abortion deaths and injuries are well-known, especially expanding access to effective modern methods of contraception and to safe abortion. The three countries highlighted in this paper have all managed to make considerable progress in implementing many of these solutions, in spite of limited resources. Romania and South Africa also underscore the rapid improvements possible in women's health once safe abortion becomes widely available. The evidence is compelling. For many countries that have yet to act, the political commitment to alter the current landscape of preventable deaths from abortion awaits.

## Competing interests

The authors declare that they have no competing interests.

## Authors' contributions

JB conceptualized the study and contributed to the writing, research, data analysis, editing and coordination of the paper. KA contributed to the research, methodological design, data analysis and editing of the paper. GS contributed to the research, data analysis, writing and editing of the paper.

All authors have read and approved the final version of the paper.
